# Pair Natural Orbitals for Coupled Cluster Quadratic Response Theory

**DOI:** 10.1021/acs.jpca.5c01617

**Published:** 2025-05-09

**Authors:** Jose P. Madriaga, Monika Kodrycka, T. Daniel Crawford

**Affiliations:** Department of Chemistry, 1757 Virginia Tech, Blacksburg, Virginia 24061, United States

## Abstract

Reduced-scaling approaches have yielded significant improvements in the computational efficiency of coupled cluster methods, making them more feasible for studying large molecules. In this work, we extend the use of pair natural orbitals (PNOs) to frequency-dependent quadratic response properties. We evaluate the performance of PNOs alongside methods optimized for response properties that derive from an approximate field-perturbed density matrix known as perturbation-aware PNOs (PNO++). Additionally, we concatenate the PNO and PNO++ spaces to obtain the combined-PNO++ method, which is tailored to simultaneously maintain the accuracy of the CCSD correlation energies and response properties. We analyze the truncation errors associated with these methods using first electric dipole hyperpolarizability – specifically the average second-harmonic generation and optical refractivity, using canonical coupled cluster singles and doubles (CCSD) as a reference. The performance analysis of the PNO family provides valuable insights into the viability of implementing CCSD quadratic response properties at a full-production level, highlighting which techniques may yield the most successful results.

## Graphical abstract

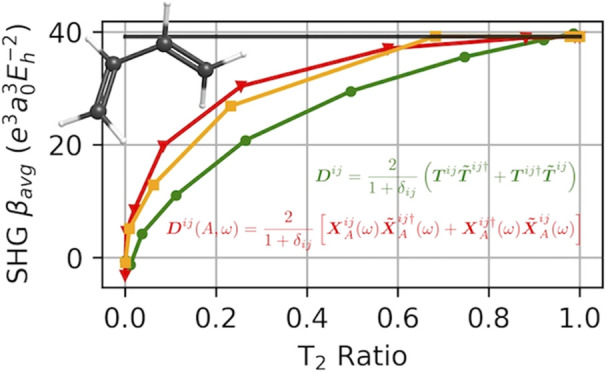

## 1 Introduction

The study of nonlinear optical (NLO) properties of molecules and materials has grown dramatically since the first reported observation of second-harmonic generation by Franken,^1^ seven decades ago. Our ever-developing understanding of light-matter interactions has paved the way for advancing new technologies^2^ driven by NLO processes to amplify and/or produce diverse signals related to information technology, scientific instrumentation, medicine, *etc.* For example, three-dimensional data memory storage^3^ takes advantage of a two-photon phenomenon that nonlinearly excites states of molecules embedded within a material to “etch” information. Another example is second-harmonic generation microscopy,^4^ which is a noninvasive imaging tool for observing noncentrosymmetric structures, e.g., collagen fibers, in living specimens. Such advances contribute to the growing demand for robust and reliable quantum mechanical methods capable of accurately predicting higher-order molecular responses to both electric and magnetic fields.

A widely used approach for simulating NLO properties is response theory (or time-dependent perturbation theory), which describes the interaction of the molecular electronic structure with weak electromagnetic fields.^5–13^ Based on expansion of the wave function in terms of appropriate multipole operators/fields, one can associate components in each perturbational order with molecular observables. For example, second-harmonic generation is a component of the perturbative expansion of the time-dependent expectation value of the electric dipole operator to the second order in external dynamic electric fields (the quadratic response function). Other response functions provide routes to frequency-dependent polarizabilities/magnetizabilities, hyperpolarizabilities, and mixed electric-/magnetic-field properties.^14–24^

While numerous methods exist for modeling quadratic response,^25–36^ coupled cluster (CC) theory,^37–46^ in particular, provides one of the most robust and systematically improvable descriptions of dynamic electron correlation, often yielding highly accurate predictions of a wide range of molecular properties. However, routine CC calculations are limited to relatively small systems due to their steep computational cost [e.g., $O\left(N^{6}\right)$ for CC singles and doubles (CCSD), where *N* represents the size of the system]. This high-degree polynomial scaling arises from the use of nonlocalized, canonical molecular orbitals (MOs) used to formulate the underlying determinantal structure of the CC wave function.

Many attempts have been made to reduce this high-degree polynomial scaling based on orbital localization,^47–53^ tensor decomposition,^54–58^ and fragmentation schemes.^59–62^ For the former, the assumption is made that electron correlation is a relatively short-range phenomenon related to molecules and materials with large HOMO–LUMO gaps (insulators), and thus, a localized MO structure can introduce greater sparsity in the resulting wave function, thereby reducing the computational cost. Several such local-correlation/reduced-scaling approaches have been used, such as the projected-atomic-orbital (PAO) approach proposed by Pulay and Sæbø,^47,63–65^ and applied to the coupled cluster framework by Werner, Schütz, and co-workers.^50,66–70^ Another example is the pair natural orbital (PNO) approach, which was originally proposed by Edmiston and Krauss^71^ and further explored by Ahlrichs et al.^72^ and Meyer.^73^ However, this method did not initially gain wide acceptance until 2009 when pioneering work by Neese et al.^49,74^ streamlined the integral transformation step, which had been the primary bottleneck, using density-fitting methods and integral screening. Subsequent major improvements from Neese and co-workers, including expansion of the PNOs in PAOs rather than canonical virtual MOs to yield the domain-based local PNO-CC (DLPNO-CC) method,^75,76^ the development of the SparseMaps formulation, which enables general, linear-scaling algorithms that take advantage of data sparsity,^77–80^ and more. As local-correlation/reduced-scaling methods have proven their worth over time, many researchers have begun intertwining their schematics and further improvements in an impressive array of existing quantum chemical frameworks.^51–53,62,75,79–96^

The focus of the present work is the PNO approach, in which compact virtual-MO subspaces are constructed for each unique pair of occupied MOs using an approximate correlated one-electron density (typically taken from second-order perturbation theory). This yields high levels of predictable sparsity in the resulting ground-state CC wave function and has been shown to provide excellent recovery of correlation energies and related properties.^97–99^ However, for field-dependent response properties, such as polarizabilities, optical rotations, etc., PNO–CC methods can produce qualitative errors.^100,101^ Attempts to overcome these problems include the use of field-perturbed densities to define the correlation domains, leading to the “perturbation-aware” PNO++ and “combined” PNO++ approaches developed in our group.^95,96,102^ In our previous studies, we observed significant improvement of PNO++ and CPNO++ over their PNO counterparts for linear response properties such as dynamic polarizabilities and specific rotations, even after truncating the correlation space by more than 50%. Thus, the question arises as to how far these techniques can be applied to higher-order properties and which technique would result in a more accurate prediction while reducing as much of the wave function space as possible. The purpose of this work is to provide a detailed performance analysis of the different PNO schemes for the CCSD quadratic response (QR) function – specifically second-harmonic generation (SHG) and optical refractivity (OR), on a number of molecular test cases to determine the extent of wave function truncation possible while still maintaining the accuracy of the canonical CCSD response approach.

## 2 Theory

### 2.1 Coupled Cluster Quadratic Response

Coupled cluster response theory is an accurate way to simulate the response of a system to an external electromagnetic field, with the time-dependent field treated as a perturbation. Adapting from Koch and Jørgensen,^15,16^ the coupled cluster quadratic response function is the second-order term in the perturbative expansion of the time-independent operator *Â* in response to both external field *B̂* and external field *Ĉ* and can be expressed in terms of perturbed wave function operators, dependent on the frequency ω_1_ of external field *B̂* and ω_2_ of external field *Ĉ*:

1 $${\left\langle \left\langle Â;\mathrm{B̂},Ĉ\right\rangle \right\rangle }_{\omega_{1},\omega_{2}}=\frac{1}{2}\mathrm{P̂}\left(\omega_{1},\omega_{2}\right)\left(\left\langle 0\right|\left({Ŷ}^{B}\left(\omega_{1}\right)\left[\mathrm{A̅},{\mathrm{X̂}}^{C}\left(\omega_{2}\right)\right]+{Ŷ}^{C}\left(\omega_{2}\right)\left[\mathrm{A̅},{\mathrm{X̂}}^{B}\left(\omega_{1}\right)\right)+{Ŷ}^{A}\left(-\omega_{1}-\omega_{2}\right)\left[\mathrm{B̅},{\mathrm{X̂}}^{C}\left(\omega_{2}\right)\right]+{Ŷ}^{A}\left(-\omega_{1}-\omega_{2}\right)\left[\mathrm{C̅},{\mathrm{X̂}}^{B}\left(\omega_{1}\right)\right]+{Ŷ}^{A}\left(-\omega_{1}-\omega_{2}\right)\left[\left[\mathrm{H̅},{\mathrm{X̂}}^{B}\left(\omega_{1}\right)\right],{\mathrm{X̂}}^{C}\left(\omega_{2}\right)\right]+\left(1+\mathrm{\Lambda ̂}\right]\left[\left[\mathrm{A̅},{\mathrm{X̂}}^{B}\left(\omega_{1}\right)\right],{\mathrm{X̂}}^{C}\left(\omega_{2}\right)\right]+\eta^{\mathrm{ABC}}\left(\omega_{1},\omega_{2}\right)\right)\left|0\right\rangle \right)$$

where

2 $$\eta^{\mathrm{ABC}}\left(\omega_{1},\omega_{2}\right)=\left(1+\mathrm{\Lambda ̂}\right)\left[\left[\mathrm{B̅},{\mathrm{X̂}}^{A}\left(-\omega_{1}-\omega_{2}\right)\right],{\mathrm{X̂}}^{C}\left(\omega_{2}\right)\right]+\left(1+\mathrm{\Lambda ̂}\right)\left[\left[\mathrm{C̅},{\mathrm{X̂}}^{A}\left(-\omega_{1}-\omega_{2}\right)\right],{\mathrm{X̂}}^{B}\left(\omega_{1}\right)\right]+{Ŷ}^{B}\left(\omega_{1}\right)\left[\left[\mathrm{H̅},{\mathrm{X̂}}^{A}\left(-\omega_{1}-\omega_{2}\right)\right],{\mathrm{X̂}}^{C}\left(\omega_{2}\right)\right]+{Ŷ}^{C}\left(\omega_{2}\right)\left[\left[\mathrm{H̅},{\mathrm{X̂}}^{A}\left(-\omega_{1}-\omega_{2}\right)\right],{\mathrm{X̂}}^{B}\left(\omega_{1}\right)\right]+\left(1+\mathrm{\Lambda ̂}\right)\left[\left[\left[\mathrm{H̅},{\mathrm{X̂}}^{A}\left(-\omega_{1}-\omega_{2}\right)\right],{\mathrm{X̂}}^{B}\left(\omega_{1}\right)\right],{\mathrm{X̂}}^{C}\left(\omega_{2}\right)\right]+{Ŷ}^{B}\left(\omega_{1}\right)\left[\mathrm{C̅},{\mathrm{X̂}}^{A}\left(-\omega_{1}-\omega_{2}\right)\right]+{Ŷ}^{C}\left(\omega_{2}\right)\left[\mathrm{B̅},{\mathrm{X̂}}^{A}\left(-\omega_{1}-\omega_{2}\right)\right]$$

where the permutation operator *P̂*(ω_1_, ω_2_) is defined as

3 $$\mathrm{P̂}\left(\omega_{1},\omega_{2}\right)f\left(\omega_{1},\omega_{2}\right)=f\left(\omega_{1},\omega_{2}\right)+f\left(-\omega_{1},-\omega_{2}\right)$$

The *A̅* is a similarity-transformed operator analogous to the similarity-transformed Hamiltonian, *e* ^–*T̂*^ *Ĥe* ^*T̂*^, or *H̅*, while the frequency-dependent perturbed operators *X̂* ^*A*^(ω) and *Ŷ* ^*A*^(ω) for operator *Â* (*B̂* and *Ĉ* respectively) are computed using a set of perturbed wave functions equations:

4 $$\left\langle \mu \right|\left[\mathrm{H̅},{\mathrm{X̂}}^{A}\left(\omega \right)\right]-\omega {\mathrm{X̂}}^{A}\left(\omega \right)\left|0\right\rangle =-\left\langle \mu \right|\mathrm{A̅}\left|0\right\rangle$$

and

5 $$\left\langle 0\right|{Ŷ}^{A}\left(\omega \right)\left[\mathrm{H̅},{\mathrm{\tau ̂}}_{\mu }\right]+\omega \left[{Ŷ}^{A}\left(\omega \right),\tau_{\mu }\right]\left|0\right\rangle =-\left\langle 0\right|\left(1+\mathrm{\Lambda ̂}\right)\left[\mathrm{A̅},{\mathrm{\tau ̂}}_{\mu }\right]+\left[\left[\mathrm{H̅},{\mathrm{X̂}}^{A}\right],{\mathrm{\tau ̂}}_{\mu }\right]\left|0\right\rangle$$

where Λ̂ is the left-hand coupled cluster amplitude and τ̂_μ_ is a second-quantized excitation operator. The right-hand amplitudes are solved first, and subsequently, they are plugged into the left-hand amplitude equations as inhomogeneous terms. It needs to be emphasized that these equations must be solved for every Cartesian direction of the given perturbation operators, including both positive and negative frequencies. Once we assume that operators *Â*, *B̂*, and *Ĉ* in eq 1 are the electric dipole operators, the first hyperpolarizability is defined as a third-rank tensor with 3^3^ = 27 components:

6 $$\sum_{\mathrm{ijk}}\beta_{\mathrm{ijk}}=\langle \left\langle {\mathrm{\mu ̂}}_{i};{\mathrm{\mu ̂}}_{j},{\mathrm{\mu ̂}}_{k}\right\rangle \rangle_{\omega_{1},\omega_{2}}$$

where the indices *i*, *j*, *k* are Cartesian directions. We note that, while the first hyperpolarizability is a third-rank tensor, only certain pieces are needed for computing frequency-dependent response properties, e.g., SHG and OR require seven, as can be seen in eq 21 below, with additional symmetry based on the chosen system coordinates.

### 2.2 Pair Natural Orbitals

In the local pair natural orbital (LPNO) approach,^74^ the occupied MOs are first localized using standard methods such as that suggested by Pipek–Mezey^103^ or Boys.^104^ Next, for each pair of occupied orbitals, *ij*, a compact set of virtual PNOs ({ *a̅*_*ij*_}) is obtained using a unitary transformation,

7 $$\left|{\mathrm{a̅}}_{\mathrm{ij}}\right\rangle =\sum_{a}Q_{\mathrm{a̅}a}^{\mathrm{ij}}\left|a\right\rangle$$

where the matrix *Q*_*a̅a*_^*ij*^ is comprised of the eigenvectors of the pairwise virtual–virtual block of an approximate one-electron density matrix,

8 $$D^{\mathrm{ij}}Q^{\mathrm{ij}}=n^{\mathrm{ij}}Q^{\mathrm{ij}}$$

In the most common LPNO-CC implementations, ***D*** ^*ij*^ is constructed from the doubles amplitudes, ***T*** ^*ij*^, from the first-order wave function of many-body perturbation theory (MBPT),

9 $$D^{\mathrm{ij}}=\frac{2}{1+\delta_{\mathrm{ij}}}\left(T^{\mathrm{ij}}{\mathrm{T̃}}^{\mathrm{ij}†}+T^{\mathrm{ij}†}{\mathrm{T̃}}^{\mathrm{ij}}\right)$$

where

10 $${\mathrm{T̃}}^{\mathrm{ij}}=2T^{\mathrm{ij}}-T^{\mathrm{ij}†}$$

and the MOs are assumed to be spin-restricted. In eq 8, the eigenvalues, ***n*** ^*ij*^, are occupation numbers that are assumed to identify the most significant virtual PNOs for each pair, *ij*, for recovering the correlation energy. Thus, the size of the virtual space/correlation domain for the given pair is reduced by removing all PNOs with occupation numbers falling below a predetermined threshold, *T*_PNOcutoff_. In addition, the virtual-PNO components of the energy denominator needed to update the wave function amplitudes in each iteration are obtained by first transforming the virtual-MO block of the Fock matrix to the PNO space,

11 $$F_{\mathrm{a̅}\mathrm{b̅}}^{\mathrm{ij}}=\sum_{\mathrm{ab}}Q_{\mathrm{a̅}a}^{\mathrm{ij}}F_{\mathrm{ab}}{\left(Q_{\mathrm{b̅}b}^{\mathrm{ij}}\right)}^{†}$$

and then transforming the result to the semicanonical basis for the given pair,

12 $$F^{\mathrm{ij}}L^{\mathrm{ij}}=\epsilon^{\mathrm{ij}}L^{\mathrm{ij}}$$

For the related PNO++ method,^95,96^ which is intended to obtain molecular response properties, the first-order (field) perturbed MBPT density is used analogously to the PNO pair density [eq 9],

13 $$D^{\mathrm{ij}}\left(A,\omega \right)=\frac{2}{1+\delta_{\mathrm{ij}}}\left[X_{A}^{\mathrm{ij}}\left(\omega \right){\mathrm{X̃}}_{A}^{\mathrm{ij}†}\left(\omega \right)+X_{A}^{\mathrm{ij}†}\left(\omega \right){\mathrm{X̃}}_{A}^{\mathrm{ij}}\left(\omega \right)\right]$$

with ***X*** *~*_*A*_^*ij*^(ω) defined similarly to eq 10. To compute the perturbed densities from MP2-level calculations, one needs to specify the first-order perturbed guess amplitudes *X*_*ab*_^*ij*^(*A*, ω), employing a particular perturbation’s matrix elements, *A̅*_*ab*_^*ij*^, and Hamiltonian matrix elements *H̅*_*pp*_ (both similarity-transformed):

14 $$X_{\mathrm{ab}}^{\mathrm{ij}}\left(A,\omega \right)=\frac{{\mathrm{A̅}}_{\mathrm{ab}}^{\mathrm{ij}}}{{\mathrm{H̅}}_{\mathrm{ii}}+{\mathrm{H̅}}_{\mathrm{jj}}-{\mathrm{H̅}}_{\mathrm{aa}}-{\mathrm{H̅}}_{\mathrm{bb}}+\omega }$$

The Hamiltonian matrix elements are given by

15 $${\mathrm{H̅}}_{\mathrm{ii}}=f_{\mathrm{ii}}+\sum_{\mathrm{nef}}t_{\mathrm{ef}}^{\mathrm{in}}\left(2\left\langle \mathrm{in}\right|\mathrm{ef}\rangle -\left\langle \mathrm{in}\right|\mathrm{fe}\rangle \right)$$

and

16 $${\mathrm{H̅}}_{\mathrm{aa}}=f_{\mathrm{aa}}+\sum_{\mathrm{mnf}}t_{\mathrm{fa}}^{\mathrm{mn}}\left(2\left\langle \mathrm{mn}\right|\mathrm{fa}\rangle -\left\langle \mathrm{mn}\right|\mathrm{fa}\rangle \right)$$

where *f*_*pq*_ is the Fock matrix, ⟨*pq*|*rs*⟩ denotes two-electron integrals in Dirac’s notation, and *t*_*ab*_^*ij*^ are MP2 amplitudes. Similarity-transformed Hamiltonian matrix elements are computed by applying the Baker-Campbell-Hausdorff expansion to the second-quantized Hamiltonian^105^ using only the *T̂*_2_ amplitudes. Once the perturbed pair densities ***D*** ^*ij*^(*A*, ω) are completed, we follow the same steps as the PNO scheme to obtain the PNO++ basis, but using a separately chosen cutoff, *T*_PNO++cutoff_. For the CPNO++ approach, the truncation step is based on two thresholds, T_PNOcutoff_ and T_PNO++cutoff_ for the unperturbed and perturbed densities, respectively. By providing the same cutoffs for both, we can consolidate the truncation to a single value of the form T_CPNO++cutoff_. Then the **Q**_*PNO*_^*ij*^ and **Q**_*PNO*++_^*ij*^ are then concatenated,

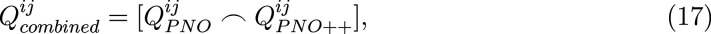

17
and a QR decomposition is used to orthogonalize the concatenated space.^95,96^

We note that for a given molecule, basis set, and choice of PNO/PNO++/CPNO++ cutoffs, we use identical pairwise virtual PNO spaces for all components entering into eq 1, including *H̅*, Λ̂, *A̅*, *X̂* ^*A*^(ω), and *Ŷ* ^*A*^(ω), to ensure equivalence between analytic- and numerical-derivative formulations of field-dependent properties, such as hyperpolarizabilities. In addition, we note that, while we would expect such truncations to yield similar sparsity (and errors) in the representations of *X̂* ^*A*^(ω) and *Ŷ* ^*A*^(ω), this does not imply that we should expect to see similar error dependence of the quadratic response function as compared to the linear response due to the order-by-order dependence of each response function of products of such truncated operators. While the linear response function involves binary products of *X̂* ^*A*^(ω) and *A̅*, the quadratic response function naturally requires products of all three *X̂* ^*A*^(ω), *Ŷ* ^*A*^(ω), and *A̅*, thus increasing its potential sensitivity to errors in each component.

## 3 Computational Details

We investigated the accuracy of the CCSD level frequency-dependent hyperpolarizability using reduced-scaling methods PNO, PNO++, and CPNO++ using PyCC, our open-source Python-based pilot program that provides a reference implementation for a range of CC methods.^106^ The code utilizes the Psi4 electronic structure package^107^ to compute the Hartree–Fock reference wave function, to localize the occupied orbitals, and to provide the requisite one- and two-electron integrals.

Starting from these input data, we carry out canonical-MO CCSD calculations to solve for the ground-state *T̂* and Λ̂ amplitudes, as well as the perturbed *X̂* ^*A*^(ω) and *Ŷ* ^*A*^(ω) amplitudes. However, in each iteration of these equations, we “filter” the nonlocal contributions to the corresponding residuals, ***R*** ^*ij*^ by transforming their canonical-MO counterparts to the semicanonical PNO basis using **Q** ^*ij*^ and **L** ^*ij*^ as

18 $${\mathrm{R̅}}^{\mathrm{ij}}=L^{\mathrm{ij}†}Q^{\mathrm{ij}†}R^{\mathrm{ij}}Q^{\mathrm{ij}}L^{\mathrm{ij}}$$

We then apply the relevant energy denominators, which consist of diagonal occupied-MO Fock matrix elements, *f*_*ii*_, and semicanonical virtual-orbital energies, ϵ̅_*a*_^*ij*^, to obtain the increment to the amplitudes in the PNO basis,

19 $${\mathrm{\Delta ̅}}_{\mathrm{ab}}^{\mathrm{ij}}=\frac{{\mathrm{R̅}}_{\mathrm{ab}}^{\mathrm{ij}}}{f_{\mathrm{ii}}+f_{\mathrm{jj}}-{\mathrm{\epsilon ̅}}_{a}^{\mathrm{ij}}-{\mathrm{\epsilon ̅}}_{b}^{\mathrm{ij}}}$$

which we then transform back to the canonical-MO basis as

20 $$\Delta^{\mathrm{ij}}=Q^{\mathrm{ij}}L^{\mathrm{ij}}{\mathrm{\Delta ̅}}^{\mathrm{ij}}L^{\mathrm{ij}†}Q^{\mathrm{ij}†}$$

Finally, we add this increment, which contains only local contributions, to the previous iteration’s cluster amplitudes to obtain the new PNO-CC *T̂*, Λ̂, *X̂* ^*A*^(ω), or *Ŷ* ^*A*^(ω) amplitudes. We repeated this process until convergence. This formulation ensures that we use the same correlation space for all components of the (perturbed) wave function, which is essential for consistency of the resulting response properties as compared with their canonical-MO counterparts.

To test the PNO++ and CPNO++ approaches for NLO responses, we selected three classes of molecular systems: helical chains of hydrogen molecules, (H_2_)_*n*_ where *n* = 4–7, hydrogen peroxide, (*P*)-H_2_O_2_, and *cis*-1,3-butadiene, which differ in their bonding and overall electronic structure. The hydrogen-molecule chains are useful test cases in that they represent a “best-case scenario” for localization methods because the component MOs are highly localizable, and intermolecular contributions can be controlled by varying the distance between the H_2_ units, if desired. Hydrogen peroxide, on the other hand, has a somewhat more complex electronic structure and a comparable hyperpolarizability to the longest H_2_ chain considered here, (H_2_)_7_. Finally, butadiene presents another electronic structure paradigm in that the largest responses to the external electric field arise from its conjugated π MOs. (The geometry for each molecule is provided in the Supporting Information.)

For each test case, we computed the SHG (ω_1_ = ω_2_) and OR (ω_1_ = −ω_2_) using the QR function, eq 6. For simplicity, the notation for SHG and OR can be rewritten as β_*αβγ*_(−2ω, ω, ω) and β_*αβγ*_(0, −ω, ω), respectively. The reported values in this work are the hyperpolarizability average,^43,108^

21 $$\beta_{\mathrm{avg}}=\frac{1}{5}\sum_{i}\left(\beta_{\mathrm{iiz}}+\beta_{\mathrm{izi}}+\beta_{\mathrm{zii}}\right)$$

where *i* ∈ {*x*, *y*, *z*}. We note that the β_avg_ values reported here were not obtained with the molecular dipole aligned with the *z*-axis, which would be necessary for comparison to experimental measurements of the SHG and OR hyperpolarizabilities. The purpose of the present work is not to make such a comparison but instead to examine the impact of the definition and truncation of the PNO space on the quadratic response function for electric dipole fields. The use of β_*avg*_ above thus provides us with a single value to perform such an analysis.

We carried out canonical-MO and PNO-CCSD for both SHG and OR, with the latter using values of cutoffs *T*_PNOcutoff_, *T*_PNO++ cutoff_, and *T*_CPNO++ cutoff_ ranging from 10^–3^ to 0 (equivalent to canonical results). In addition, we employed convergence criteria of 10^–8^ for all Hartree–Fock SCF and CCSD iterative procedures in root-mean-squared density changes or amplitude increments. We chose a value of ω = 0.0656 au (corresponding to a wavelength of 694.3 nm) using the correlation-consistent double-ζ basis sets of Dunning Jr. and co-workers^109–111^ with diffuse functions (aug-cc-pVDZ), as well as an additional set of calculations for (H_2_)_4_ and H_2_O_2_ using the triple-ζ aug-cc-pVTZ basis. The 1*s* core orbitals on carbon and oxygen were kept frozen in all of the calculations.

## 4 Results

We have computed the second-harmonic generation (SHG) and optical refractivity (OR) first hyperpolarizabilities using PNO, PNO++, and CPNO++ in the aug-cc-pVDZ (adz) basis across a range of values of *T*_PNO cutoff_, *T*_PNO++ cutoff_, and *T*_CPNO++ cutoff_ for comparison to the canonical-MO result. To estimate the computational efficiency (i.e., relative reduction of the PNO virtual space) of each method, we plot these hyperpolarizabilities as a function of the ratio of the number of double-excitation wave function amplitudes remaining in the truncated correlation space to the number required for an untruncated calculation (the *T*_2_ ratio). Our metric for the performance of each method is the lowest cutoff for which the error in the truncated result is within 95% of that of its canonical counterpart. Given that the trends in our performance analysis of both SHG and OR are similar, we choose to focus here primarily on the former, while readers may find the latter in the Supporting Information as well as tabulation of the results discussed below in Table S1.

### 4.1 Performance Analysis

For the helical chains of hydrogen molecules, (H_2_)_*n*_ where *n* = 4 – 7, we focus on the SHG β_*avg*_ of the longest case, (H_2_)_7_, as a representative of these systems. Data for (H_2_)_4_ through (H_2_)_6_ can be found in Supporting Information. In Figure 1, we plot the SHG β_avg_ values against the *T*_2_ ratio based on a chosen cutoff for each method. The starting cutoff is 10^–3^, and each kink (with markers) along the PNO, PNO++, and CPNO++ line indicates a tighter cutoff by an order of magnitude until convergence to the CCSD canonical-MO value of SHG β_*avg*_ of −18.949 a.u., which is marked by the solid black line. For the PNO-CCSD method, we observe slow convergence, requiring a *T*_PNOcutoff_ of 10^–17^ to finally reach the canonical value, whereas the convergence is faster for PNO++ and CPNO++, requiring 10^–14^ and 10^–11^, respectively. (The relationship between the T_2_ ratio and cutoffs is provided in tables in the Supporting Information, along with the data used to generate the figures.) To examine the performance of the PNO family of methods, we identified cutoffs that provide errors within approximately ± 5% of the canonical results. For PNO-CCSD, a *T*_PNO cutoff_ of 10^–11^ removes a little over a third of the wave function amplitudes (*T*_2_ ratio of 0.594) to retain 95.6% of the SHG reference value. We observe better behavior for PNO++, where a *T*_PNO++ cutoff_ of 10^–7^ reduces the amplitude space by roughly nine-tenths (*T*_2_ = 0.127) while recovering 94.3% of the canonical SHG value. A similar trend holds for CPNO++ such that a *T*_CPNO++ cutoff_ of 10^–7^ results in two-thirds (*T*_2_ = 0.338) of the wave function being removed with 98.8% accuracy. These data suggest that the PNO++ method, which is based on the use of the pairwise *derivative* density, yields compact correlation spaces for quadratic response properties just as was previously observed for linear response.^95^ The reason for the increase in the number of wave function amplitudes for CPNO++ vs PNO++ is due to the concatenation of the eigenvectors PNO pairwise density and those of the PNO++ pairwise density in order to balance the recovery of both electron correlation and the wave function response. Similar observations hold for the shorter H_2_ helical chains.

**Figure 1:**
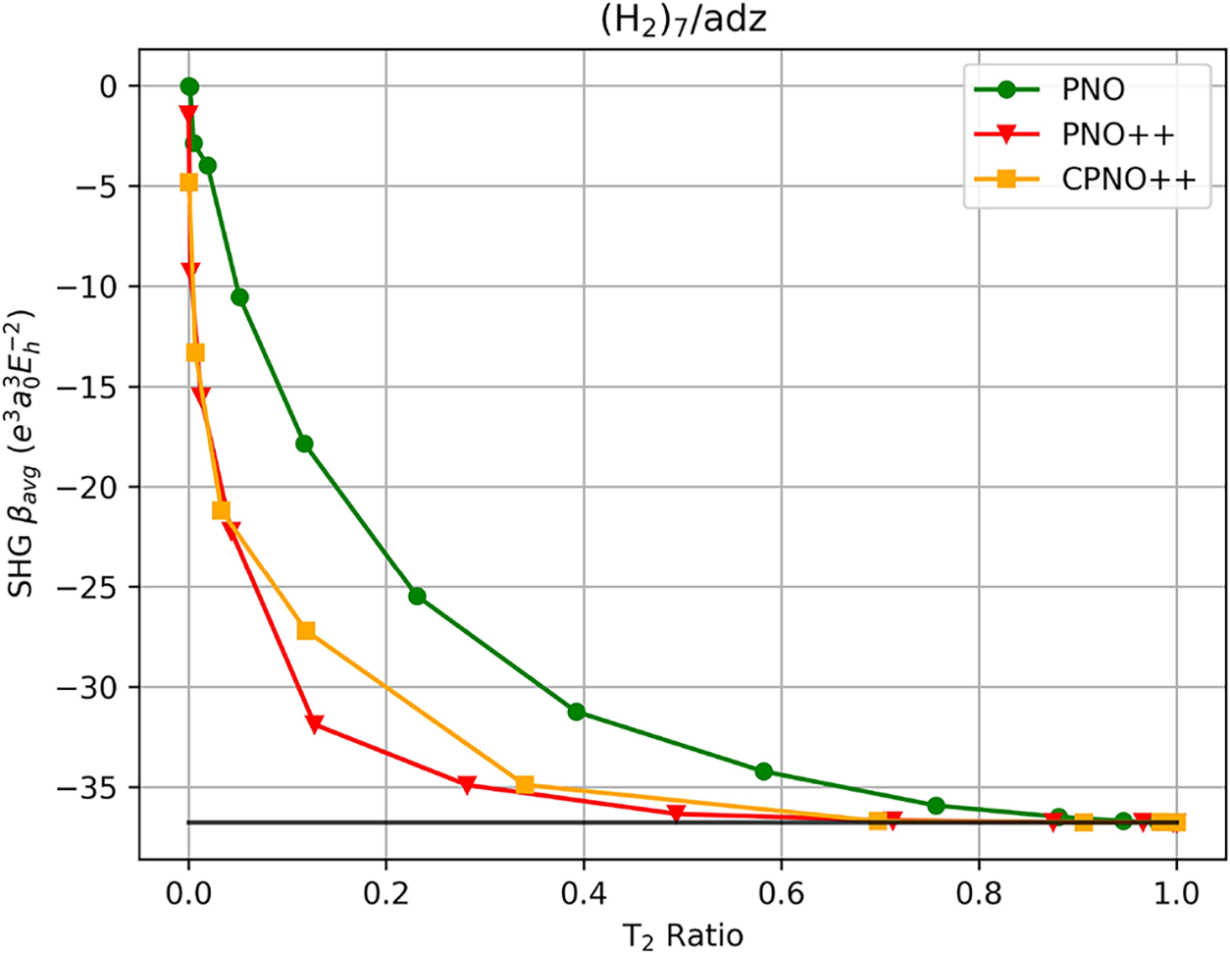
SHG first hyperpolarizability average of (H_2_)_7_ using PNO–CCSD, PNO++-CCSD, and CPNO++-CCSD in an aug-cc-pVDZ basis set as a function of the T_2_ ratio. The canonical-MO result is indicated by the solid black line.

For the second test case, hydrogen peroxide, the analysis is less straighforward compared to that of (H_2_)_7_ as illustrated in Figure 2 because of large fluctuations in the convergence behavior around the reference value for PNO++ and CPNO++. For PNO, the convergence to the reference is well-behaved even though it is slow, requiring a *T*_PNOcutoff_ of 10^–12^ to reach the canonical result vs values of *T*_PNO++cutoff_ = 10^–10^ and *T*_CPNO++cutoff_ = 10^–7^ for PNO++ and CPNO++, respectively.

**Figure 2:**
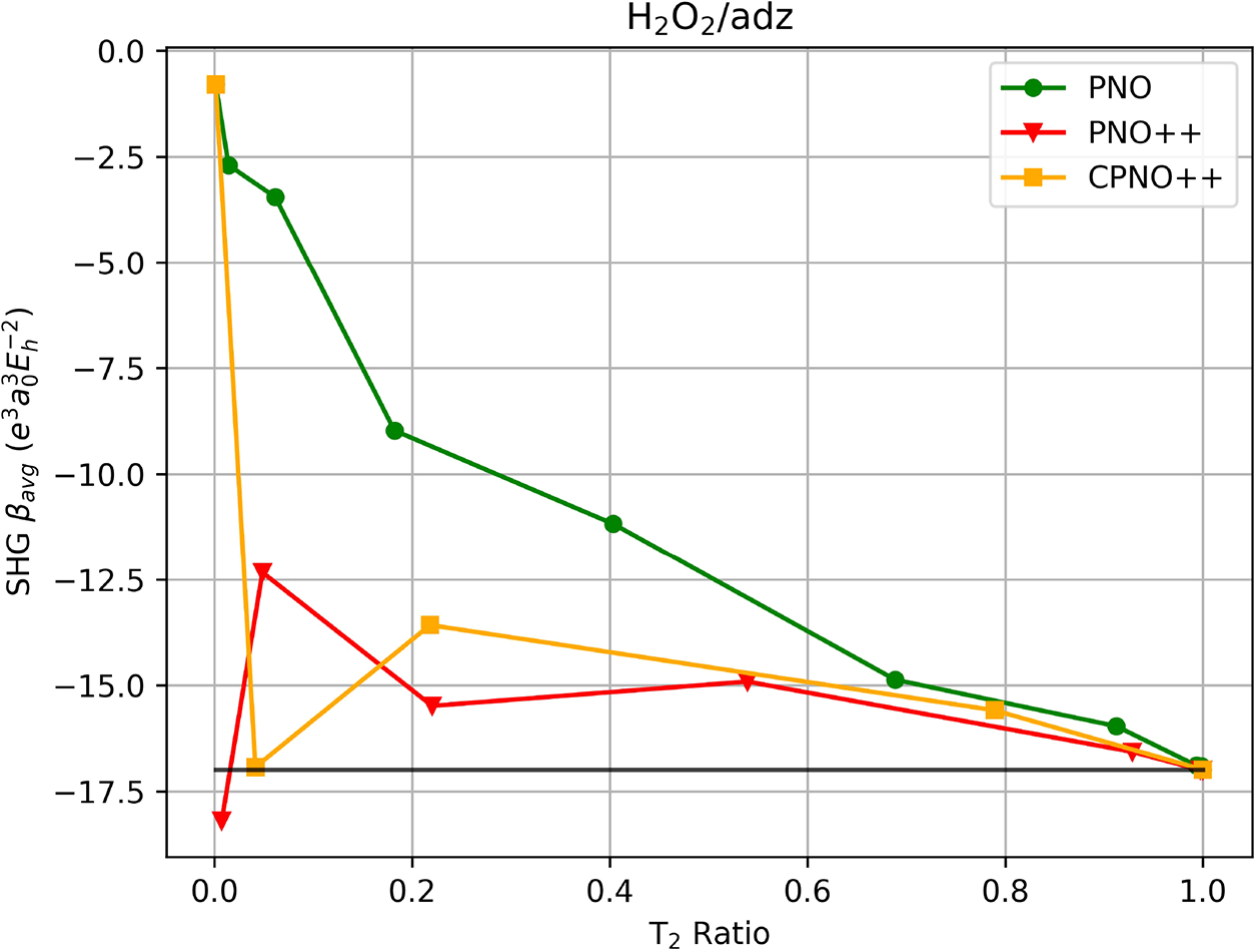
SHG first hyperpolarizability average of H_2_O_2_ using PNO–CCSD, PNO++-CCSD, and CPNO++-CCSD in an aug-cc-pVDZ basis set as a function of the T_2_ ratio. The canonical-MO result is indicated by the solid black line.

The PNO method struggles to reduce the wave function space to the desired accuracy of 95%. For a *T*_PNOcutoff_ of 10^–9^, the wave function space is only reduced by less than a tenth (*T*_2_ = 0.912) while recovering 93.9% of the canonical reference result. Tightening the cutoff to 10^–10^ allows PNO to capture 99.4%, albeit retaining essentially all of the wave function space (*T*_2_ ratio of 0.993). For PNO++ and CPNO++, the observed fluctuations allow fortuitous cancellation of errors for small cutoffs. For example, a *T*_PNO++cutoff_ of 10^–9^ captures 97.5% of the reference value PNO++, with a reduction in the wave function space by a tenth (*T*_2_ = 0.928). However, reducing *T*_PNO++cutoff_ to 10^–3^ concomitantly reduces the *T*_2_ ratio to 0.007 while overestimating the SHG β_avg_ by only 7%. Another case can be observed at a *T*_PNO++cutoff_ of 10^–5^, where we underestimate the SHG β_avg_ by roughly 9% while using less than 25% of the wave function amplitudes. Similarly, for CPNO++, the computed SHG β_*avg*_ is close to the reference at *T*_CPNO++cutoff_ of 10^–4^, resulting in a coincidental 99.6% accuracy with less than 5% of the wave function space. However, using a more reasonable *T*_CPNO++cutoff_ of 10^–6^, we capture only 91.7% of the reference with more than three-fourths of the wave function space. Clearly, such trends cannot be relied upon for predictive calculations.

In order to examine the impact of the choice of basis set on the erratic convergence behavior of the SHG average for H_2_O_2_, we have also carried out PNO, PNO++, and CPNO++ calculations using the aug-cc-pVTZ basis. In Figure 3, for PNO++/aug-cc-pVTZ, we observe fewer fluctuations in the convergence toward the reference value as compared with aug-cc-pVDZ, with such behavior occurring only at very loose cutoffs where the virtual function space is heavily truncated. For CPNO++/aug-cc-pVTZ, the convergence is well-behaved across all cutoffs examined here. Interestingly, the erratic behavior appears for PNO/aug-cc-pVTZ where it was not previously observed for the aug-cc-pVDZ case, and at small cutoffs closer to the full virtual space. However, this can be explained similarly to how PNO++/aug-cc-pVTZ oscillates rapidly for very loose cutoffs such that from *T*_PNO++cutoff_ of 10^–3^ to 10^–5^ changes the *T*_2_ ratio from 0.000005 to 0.0014 and then to 0.0109, but the SHG average fluctuates from 0.0254 to −14.167 then to −9.881. With the removal of just a couple of basis functions associated with large spatial extent (diffuse functions), which are sensitive and important to response properties. The same can be said for PNO/aug-cc-pVTZ with very tight cutoffs from *T*_PNOcutoff_ of 10^–11^ to 10^–13^ such that *T*_2_ ratio changes minutely from 0.986 to 0.9984 and then to 1.0 while a fluctuation can be observed (though the error with respect to the reference value is only around 1%, essentially noise) not as significant as PNO++ due to the basis functions (small spatial extent or inner shell functions) being removed does not play an important role in response properties and can be considered adjustments toward the full space.

**Figure 3:**
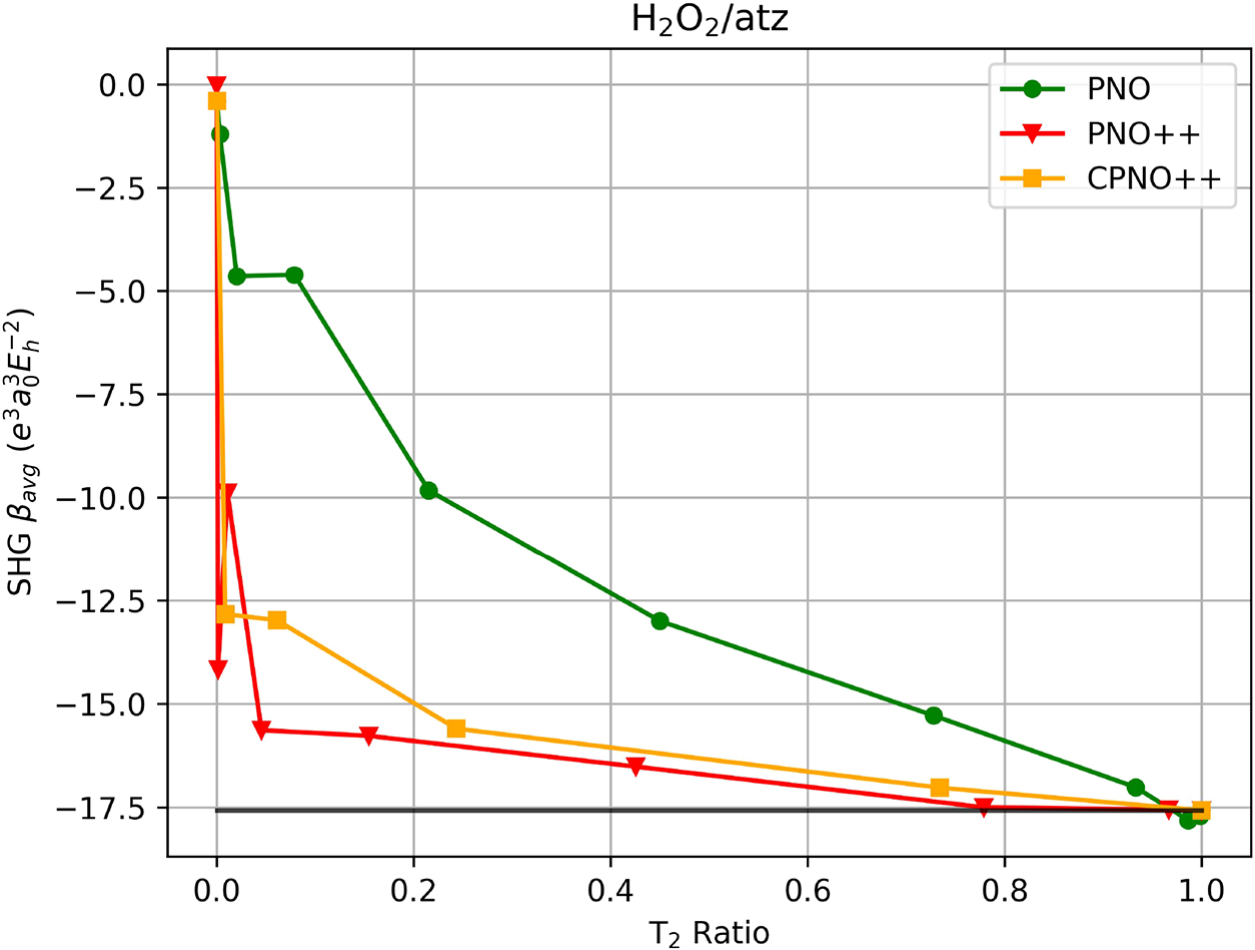
SHG first hyperpolarizability average of H_2_O_2_ using PNO–CCSD, PNO++-CCSD, and CPNO++-CCSD in an aug-cc-pVTZ basis set as a function of the T_2_ ratio. The canonical-MO result is indicated by the solid black line.

The H_2_O_2_/aug-cc-pVTZ test case provides additional information regarding the performance of the PNO family as we increase the basis set size. If we disregard the occurrence of fortuitous cancellations of errors for aug-cc-pVDZ and compare the results to the aug-cc-pVTZ case with similar levels of accuracy, then we should expect a smaller fraction of space as the basis set increases in size. For the PNO method, the aug-cc-pVTZ basis requires a *T*_PNOcutoff_ of 10^–10^ to achieve 96.8% accuracy of the SHG average with a *T*_2_ ratio of 0.933 as opposed to the aug-cc-pVDZ basis where a *T*_2_ ratio of 0.993 is needed. For PNO++, the aug-cc-pVTZ basis requires a *T*_PNO++cutoff_ of 10^–9^ to capture 99.6% of the reference with a *T*_2_ ratio of 0.779 compared to that for aug-cc-pVDZ of 0.928, while CPNO++/aug-cc-pVTZ requires a *T*_CPNO++cutoff_ of 10^–7^ to obtain 96.9% accuracy with a *T*_2_ ratio of 0.734 in compared to the aug-cc-pVDZ case of 0.789. If we lower the expected level of accuracy for PNO++ and CPNO++, we see a larger significant reduction of the space. If we choose *T*_PNO++cutoff_ of 10^–8^, we lower the accuracy to 94% with less than half the space (*T*_2_ of 0.425), and if we choose *T*_CPNO++cutoff_ of 10^–6^, we lower the accuracy to 88% with a quarter of the space (*T*_2_ of 0.243).

For the largest system examined here, *cis*-1,3-butadiene (C_4_H_6_), we observe a smoother trend compared to that of H_2_O_2_/aug-cc-pVDZ as illustrated in Figure 4. As expected, the PNO–CCSD converges slowly toward the reference, reaching the full space at *T*_PNOcutoff_ of 10^–15^. Once at *T*_PNOcutoff_ of 10^–11^, we obtain the desired accuracy of 98.5% by only removing about a tenth of the space (*T*_2_ ratio = 0.92). Lowering the *T*_PNOcutoff_ to 10^–10^, we still retain three-fourth of the space (*T*_2_ ratio = 0.747) and the accuracy is reduced to 90.7%. With the “perturbation-aware” PNO counterpart, we notice improvements such that a *T*_PNO++cutoff_ of 10^–8^ removes almost half of the wave function space (*T*_2_ ratio = 0.577) with 94.5% accuracy. Interestingly, CPNO++ does not remove as much virtual-MO space as PNO++, still retaining two-thirds of the space at *T*_CPNO++cutoff_ of 10^–7^ with a *T*_2_ ratio of 0.683 though essentially obtaining the canonical results at 39.177 au (39.207 au for CPNO++) and continues to obtain such results as it reaches the full space.

**Figure 4:**
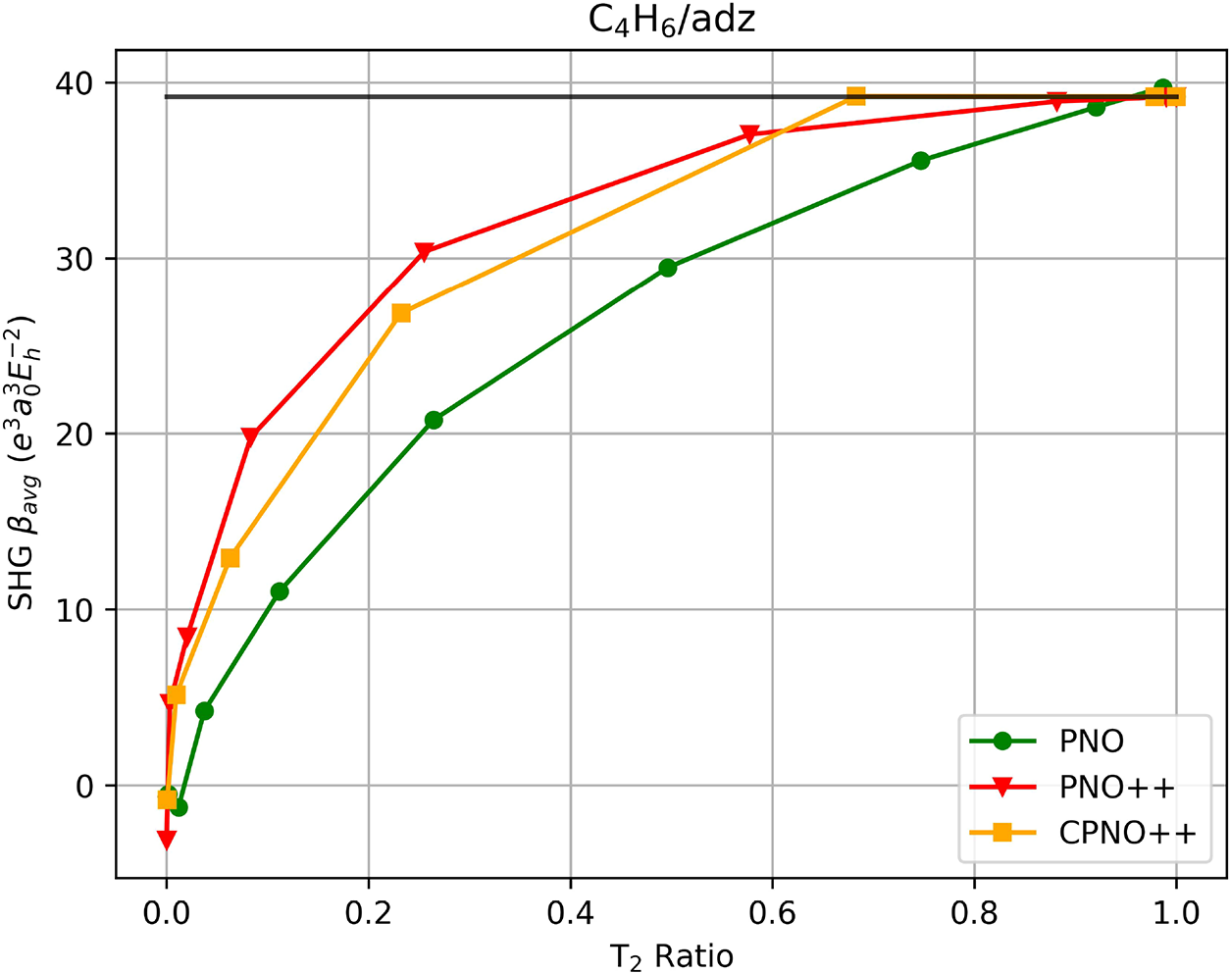
SHG first hyperpolarizability average of C_4_H_6_ using PNO–CCSD, PNO++-CCSD, and CPNO++-CCSD in an aug-cc-pVDZ basis set as a function of the T_2_ ratio. The canonical-MO result is indicated by the solid black line.

## 5 Conclusions

In this work, we examined reduced-scaling approaches based on PNOs optimized for response properties by computing truncation errors in CCSD dynamic hyperpolarizabilities for modeling second-harmonic generation and optical refractivity for a set of small molecular systems. We find that the PNO++ and CPNO++ methods share similar truncation errors and virtual-space reduction capabilities that outperform the original set of PNOs in most cases. PNO++ is observed to reduce the wave function space by as much as 87.3% while retaining roughly 95% of the canonical-MO CCSD reference value, though only for the highly localizable (H_2_)_7_ test case. For the somewhat larger molecular cases examined here, smaller reductions are observed at the same error level, e.g., 57% reduction for H_2_O_2_ (aug-cc-pVTZ) and 42.2% for butadiene. As observed in previous studies, the relative success of the PNO++ and CPNO++ methods is based on their retention of diffuse contributions to the natural orbital basis. These are essential to the accurate prediction of field-dependent properties, but they play little role in recovering dynamic electron correlation effects. Thus, while the PNO method naturally eliminates such orbitals due to their small occupation numbers, the PNO++ and CPNO++ methods weigh them more heavily through their contribution to the perturbed pair density. While this work indicates the level of truncation of the PNO/PNO++/CPNO++ pairwise virtual spaces allowed to retain an acceptable level of accuracy of the computed properties relative to the conventional, canonical-MO results, only a fully production-level implementation that includes various approximations (projected atomic orbitals, prescreening of weak pairs, etc.) as well as method-specific optimizations will provide a complete assessment of the potential computational savings possible using these methods for higher-order response properties.

## Supplementary Material



## Notes

Plots of hyperpolarizabilities for (H_2_)_*n*_, H_2_O_2_, and *cis*-1,3-butadiene for varying choices of PNOs and *T*_PNO cutoff_ are provided, along with all computed hyperpolarizability tensors for evaluating the hyperpolarizability average and its corresponding *T*_2_ Ratio. (PDF)
