# Supplementary material for: Pair Natural Orbitals for Coupled Cluster Quadratic Response Theory
Source: J Phys Chem A. 2025 May 9;129(20):4601–10. doi: 10.1021/acs.jpca.5c01617 (PMC12105027; doi:10.1021/acs.jpca.5c01617)
Supplement: Supplementary file 1 [file jp5c01617_si_001.pdf]

Supporting Information: Pair Natural Orbitals for Coupled Cluster Quadratic Response Theory

Jose P. Madriaga, Monika Kodrycka, and T. Daniel Crawford*

Department of Chemistry, Virginia Tech, Blacksburg, VA 24061, USA

E-mail: crawdad@vt.edu

Contents

1 Molecular Geometries **S2**

1.1 $(H_2)_4$ S2

1.2 $(H_2)_5$ S2

1.3 $(H_2)_6$ S2

1.4 $(H_2)_7$ S3

1.5 H_2O_2 . . . . . S3

1.6 cis-1,3,butadiene S3

2 Second Harmonic Generation **S4**

3 Optical Refractivity **S9**

References **S12**

1 Molecular Geometries

Cartesian coordinates are given in a_0.

1.1 $(H_2)_4$

```
H 0.000000 0.000000 0.000000
H 0.750000 0.000000 0.000000
H 0.000000 1.500000 0.000000
H 0.375000 1.500000 -0.649520
H 0.000000 3.000000 0.000000
H -0.375000 3.000000 -0.649520
H 0.000000 4.500000 -0.000000
H -0.750000 4.500000 -0.000000
```

1.2 $(H_2)_5$

```
H 0.000000 0.000000 0.000000
H 0.750000 0.000000 0.000000
H 0.000000 1.500000 0.000000
H 0.375000 1.500000 -0.649520
H 0.000000 3.000000 0.000000
H -0.375000 3.000000 -0.649520
H 0.000000 4.500000 -0.000000
H -0.750000 4.500000 -0.000000
H 0.000000 6.000000 -0.000000
H -0.375000 6.000000 0.649520
```

1.3 $(H_2)_6$

```
H 0.000000 0.000000 0.000000
H 0.750000 0.000000 0.000000
H 0.000000 1.500000 0.000000
H 0.375000 1.500000 -0.649520
H 0.000000 3.000000 0.000000
H -0.375000 3.000000 -0.649520
H 0.000000 4.500000 -0.000000
H -0.750000 4.500000 -0.000000
H 0.000000 6.000000 -0.000000
H -0.375000 6.000000 0.649520
H 0.000000 7.500000 -0.000000
H 0.375000 7.500000 0.649520
```

1.4 $(H_2)_7$

```
H 0.000000 0.000000 0.000000
H 0.750000 0.000000 0.000000
H 0.000000 1.500000 0.000000
H 0.375000 1.500000 -0.649520
H 0.000000 3.000000 0.000000
H -0.375000 3.000000 -0.649520
H 0.000000 4.500000 -0.000000
H -0.750000 4.500000 -0.000000
H 0.000000 6.000000 -0.000000
H -0.375000 6.000000 0.649520
H 0.000000 7.500000 -0.000000
H 0.375000 7.500000 0.649520
H 0.000000 9.000000 -0.000000
H 0.750000 9.000000 0.000000
```

1.5 H_2O_2

```
O -0.182400 -0.692195 -0.031109
O 0.182400 0.692195 -0.031109
H 0.533952 -1.077444 0.493728
H -0.533952 1.077444 0.493728
```

1.6 cis-1,3,butadiene

```
C 0.1263860 0.7363650 0.5545160
C -0.1263860 -0.7363650 0.5545160
C -0.1263860 1.5462330 -0.4896520
C 0.1263860 -1.5462330 -0.4896520
H 0.5340580 1.1553420 1.4777330
H -0.5340580 -1.1553420 1.4777330
H 0.0897160 2.6130790 -0.4556810
H -0.5704660 1.1695290 -1.4112380
H -0.0897160 -2.6130790 -0.4556810
H 0.5704660 -1.1695290 -1.4112380
```

The geometry for cis-1,3,butadiene is CCD/3-21G optimized structure provided by computational chemistry comparison and benchmark database at the National Institute of Standards and Technology.[S1]

2 Second Harmonic Generation

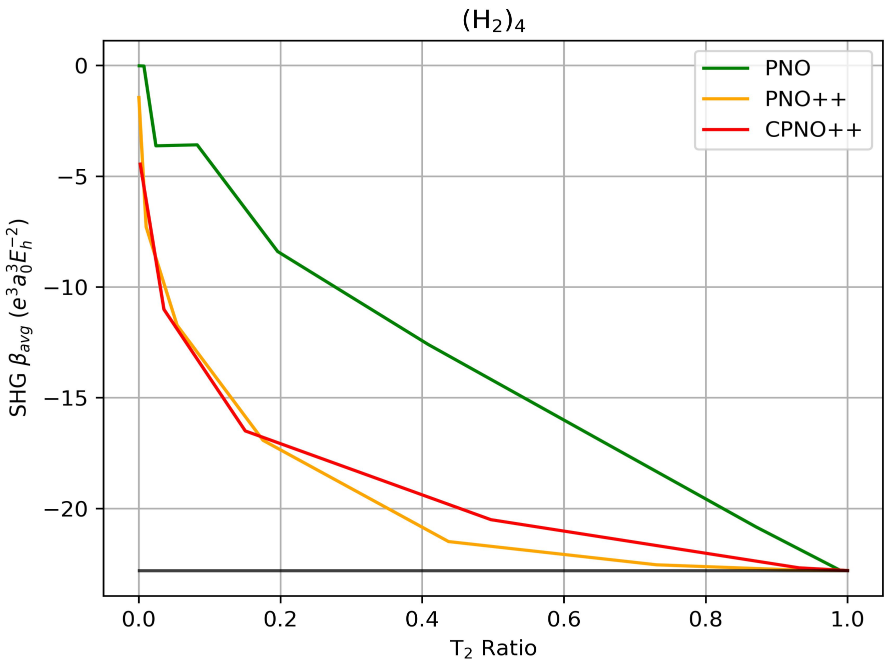

Figure S1: SHG first hyperpolarizability average of $(H_2)_4$ using LPNO-CCSD/aug-cc-pVDZ as a function of T_2 ratio

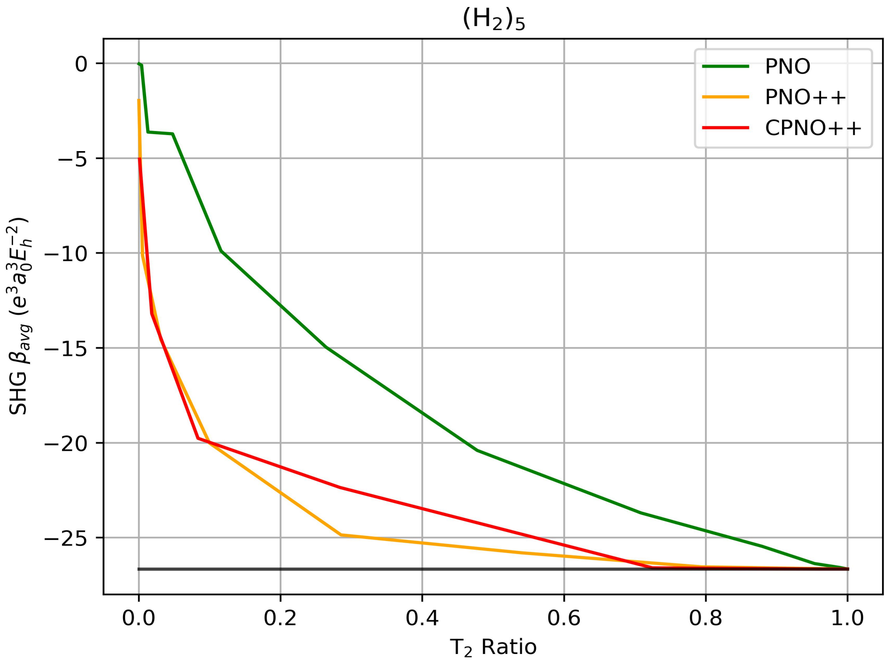

Figure S2: SHG first hyperpolarizability average of $(H_2)_5$ using LPNO-CCSD/aug-cc-pVDZ as a function of T_2 ratio

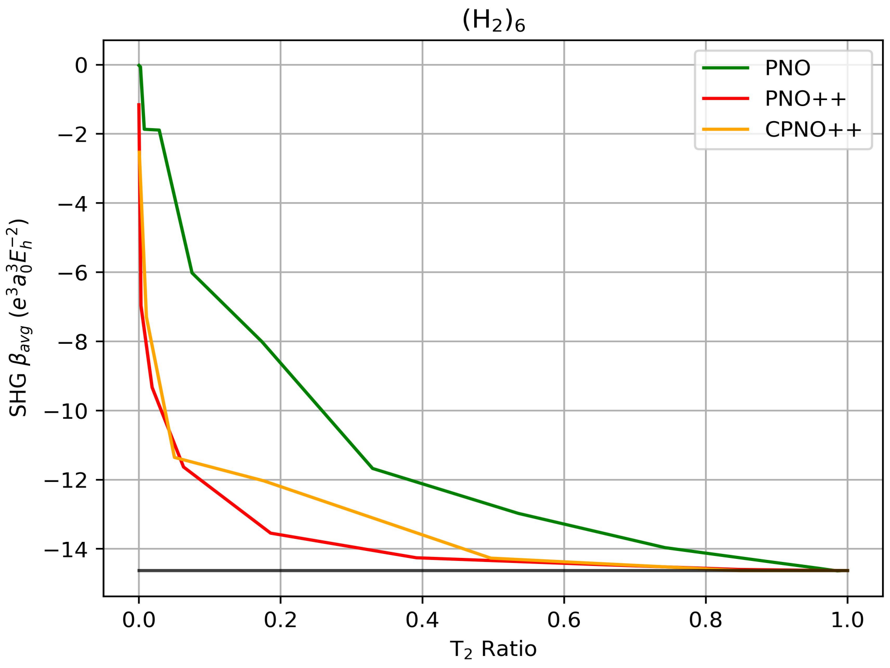

Figure S3: SHG first hyperpolarizability average of $(H_2)_6$ using LPNO-CCSD/aug-cc-pVDZ as a function of T_2 ratio

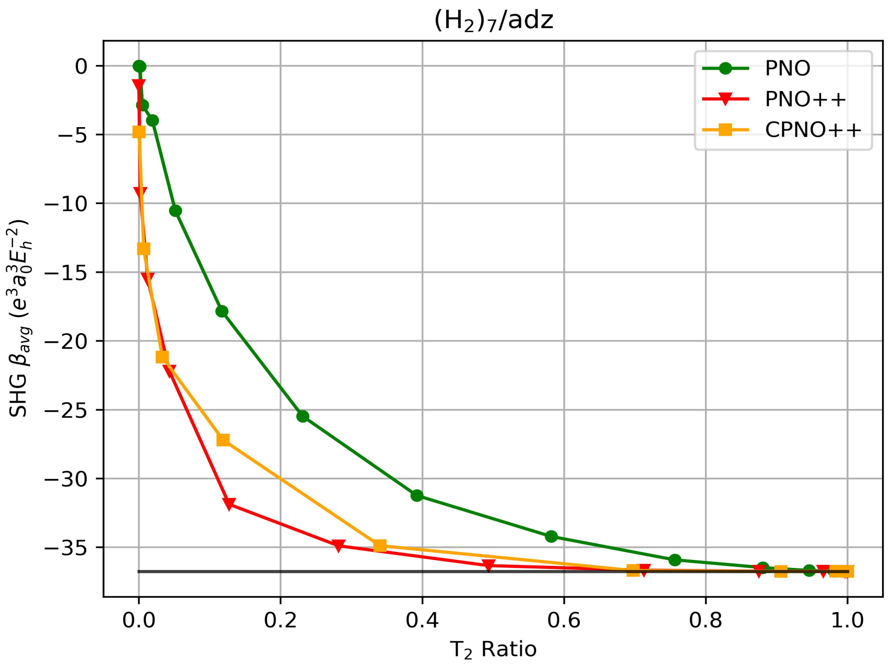

Figure S4: SHG first hyperpolarizability average of $(H_2)_7$ using LPNO-CCSD/aug-cc-pVDZ as a function of T_2 ratio

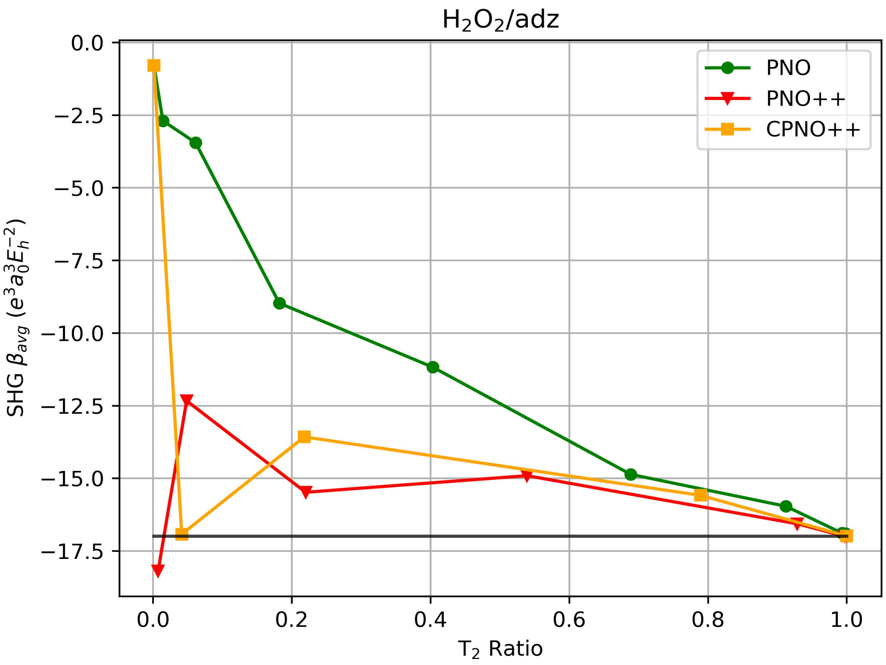

Figure S5: SHG first hyperpolarizability average of H_2O_2 using LPNO-CCSD/aug-cc-pVDZ as a function of T_2 ratio

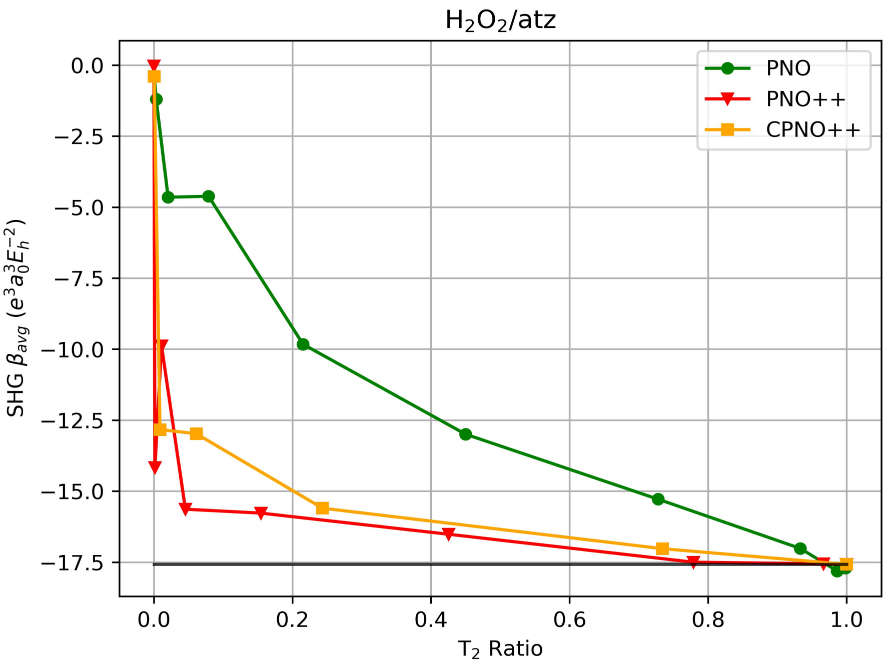

Figure S6: SHG first hyperpolarizability average of H_2O_2 using LPNO-CCSD/aug-cc-pVTZ as a function of T_2 ratio

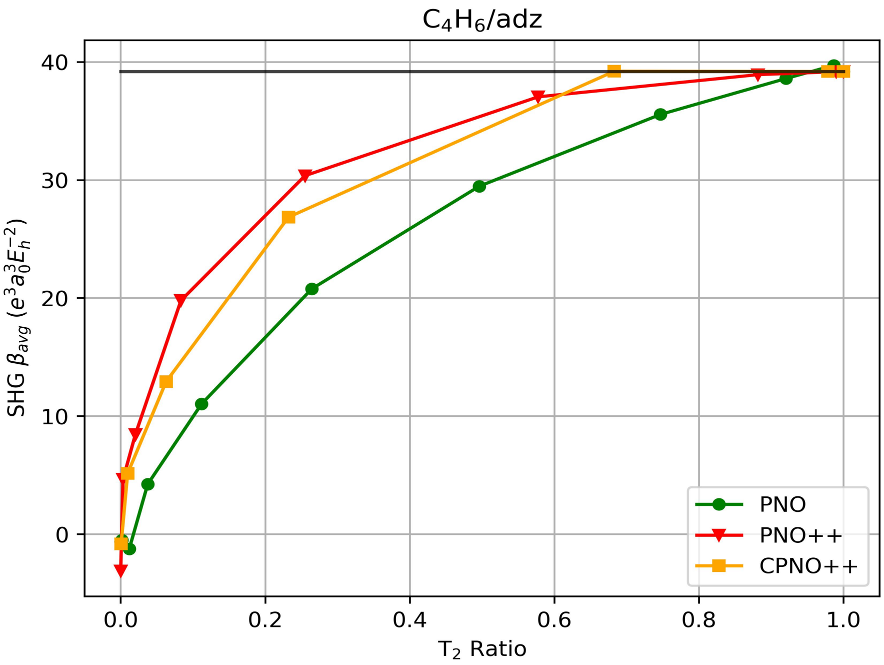

Figure S7: SHG first hyperpolarizability average of C_4H_6 using LPNO-CCSD/aug-cc-pVDZ as a function of T_2 ratio

Table S1 summarizes cutoffs, T_2 ratio, and accuracy with respect to the the Canonical CCSD SHG for the different flavors of PNO for which the error in the truncated result is within 95% of its canonical counterpart (as well as cutoffs that were highlighted in the results section [in red]) using aug-cc-pVDZ (adz) and aug-cc-pVTZ (atz).

Table S1: Performance of PNO flavors with Respect to Canonical CCSD SHG

Molecule	PNO			PNO++			CPNO++		
	Cutoff	T_2 Ratio	Accuracy	Cutoff	T_2 Ratio	Accuracy	Cutoff	T_2 Ratio	Accuracy
$(H_2)_4$/adz	10^{-10}	0.871	91.4%	10^{-6}	0.176	78.5%	10^{-6}	0.497	88.9%
	10^{-11}	0.959	93.0%	10^{-7}	0.437	94.3%	10^{-7}	0.932	99.5%
	10^{-12}	0.989	99.9%	10^{-8}	0.731	98.9%			
				10^{-9}	0.915	99.8%			
$(H_2)_5$/adz	10^{-10}	0.709	88.9%	10^{-7}	0.285	93.3%	10^{-6}	0.284	83.9%
	10^{-11}	0.880	95.5%	10^{-8}	0.541	96.8%	10^{-7}	0.725	99.8%
	10^{-12}	0.954	98.9%	10^{-9}	0.794	99.6%	10^{-8}	0.965	100%
	10^{-13}	0.989	99.7%	10^{-10}	0.932	99.9%			
$(H_2)_6$/adz	10^{-10}	0.536	88.7%	10^{-7}	0.186	92.6%	10^{-6}	0.176	82.3%
	10^{-11}	0.743	95.5%	10^{-8}	0.392	97.5%	10^{-7}	0.497	97.5%
	10^{-12}	0.886	98.2%	10^{-9}	0.648	98.8%	10^{-8}	0.849	100%
	10^{-13}	0.957	99.5%	10^{-10}	0.846	99.8%			
	10^{-14}	0.986	100%	10^{-11}	0.957	100%			
$(H_2)_7$/adz	10^{-10}	0.399	88.1%	10^{-6}	0.042	87.0%	10^{-6}	0.117	83.5%
	10^{-12}	0.776	98.3%	10^{-7}	0.127	94.4%	10^{-7}	0.338	96.3%
	10^{-13}	0.899	99.5%	10^{-8}	0.282	98.8%	10^{-8}	0.706	100%
	10^{-14}	0.956	100%	10^{-9}	0.500	99.2%			
				10^{-10}	0.729	99.6%			
H_2O_2/adz	10^{-8}	0.689	87.5%	10^{-4}	0.007	107%	10^{-6}	0.789	91.8%
	10^{-9}	0.912	94.0%	10^{-6}	0.220	91.1%	10^{-4}	0.041	99.6%
	10^{-10}	0.993	99.4%	10^{-8}	0.928	97.5%			
	10^{-11}	0.999	99.5%	10^{-9}	0.997	100%			
H_2O_2/atz	10^{-9}	0.728	87.0%	10^{-7}	0.154	89.7%	10^{-6}	0.243	88.8%
	10^{-10}	0.933	96.8%	10^{-8}	0.425	94.0%	10^{-7}	0.734	96.9%
	10^{-11}	0.986	101.3%	10^{-9}	0.779	99.6%			
C_4H_6/adz	10^{-10}	0.747	90.7%	10^{-7}	0.255	77.5%	10^{-7}	0.683	100.1%
	10^{-11}	0.921	98.5%	10^{-8}	0.578	94.5%	10^{-8}	0.978	100%
	10^{-12}	0.987	101.3%	10^{-9}	0.882	99.3%			

3 Optical Refractivity

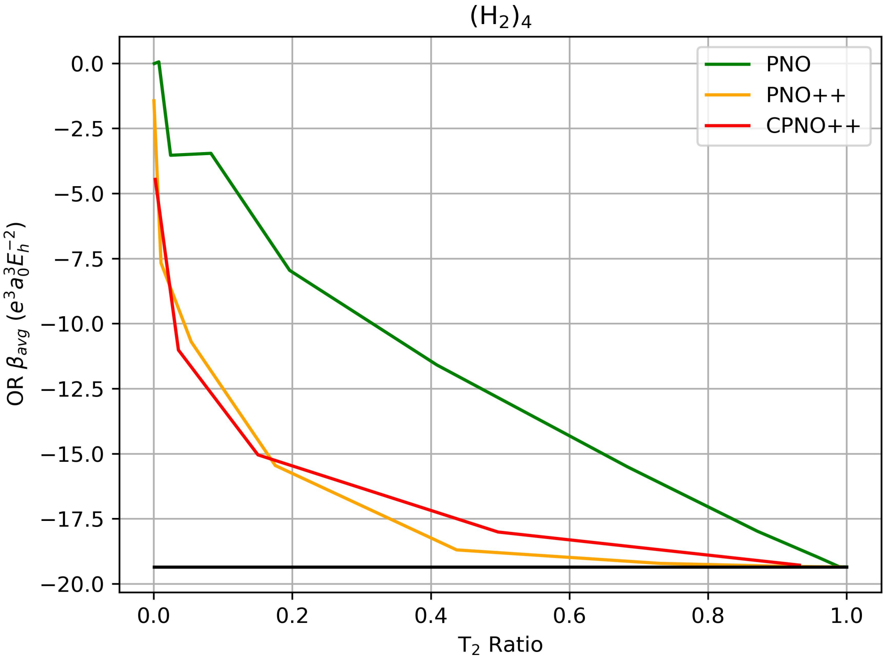

Figure S8: OR first hyperpolarizability average of $(H_2)_4$ using LPNO-CCSD/aug-cc-pVDZ as a function of T_2 ratio

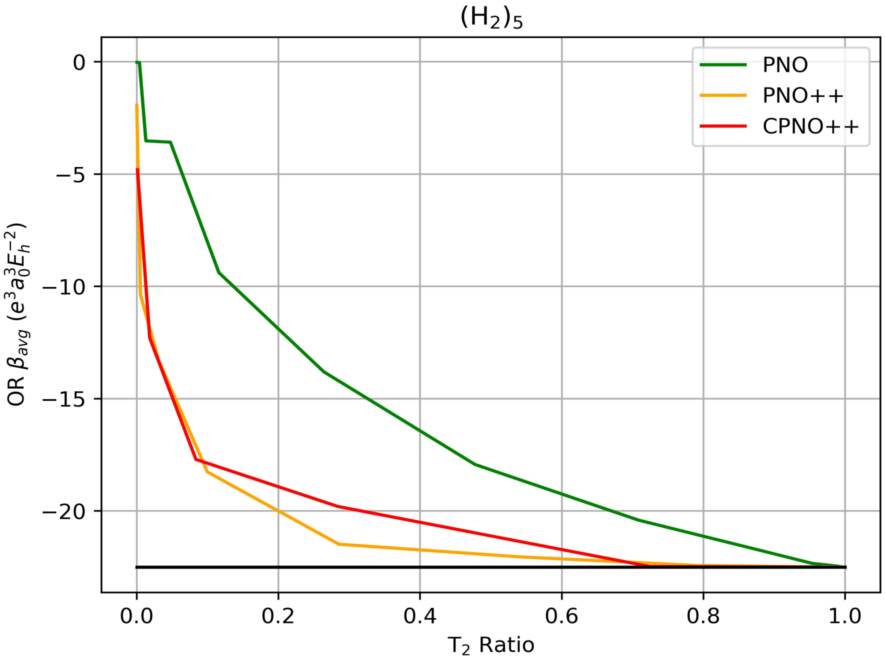

Figure S9: OR first hyperpolarizability average of $(H_2)_5$ using LPNO-CCSD/aug-cc-pVDZ as a function of T_2 ratio

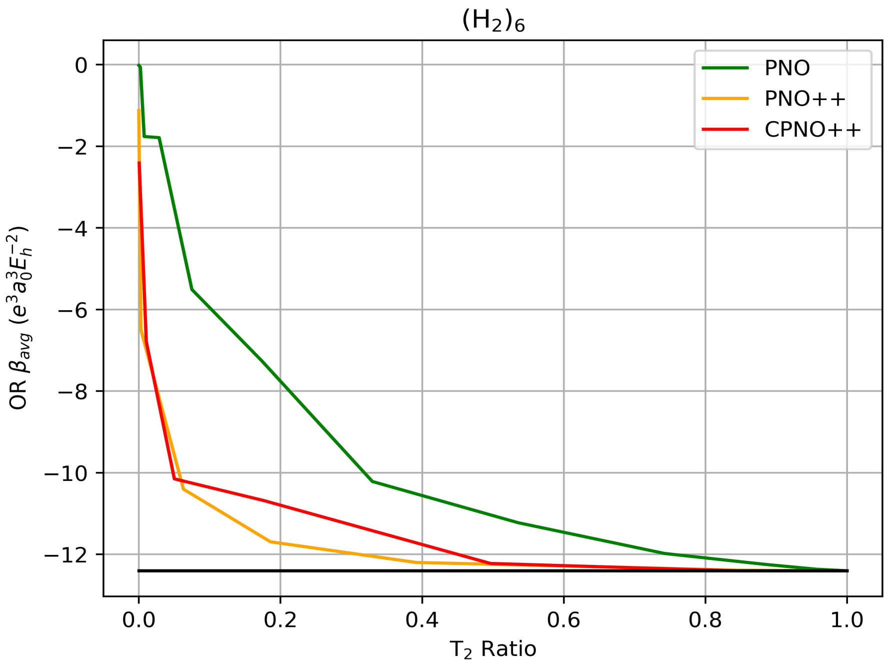

Figure S10: OR first hyperpolarizability average of $(H_2)_6$ using LPNO-CCSD/aug-cc-pVDZ as a function of T_2 ratio

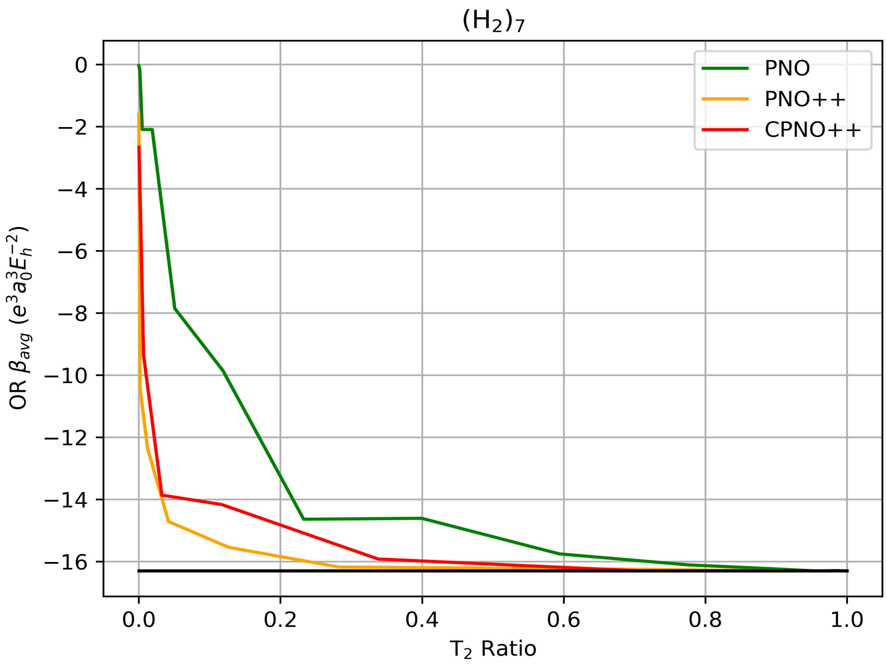

Figure S11: OR first hyperpolarizability average of $(H_2)_7$ using LPNO-CCSD/aug-cc-pVDZ as a function of T_2 ratio

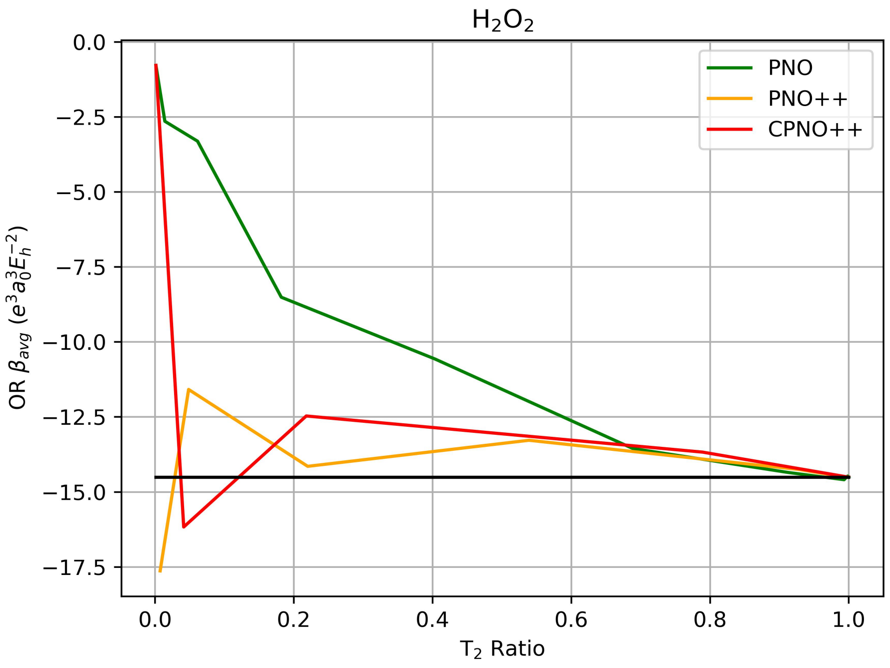

Figure S12: OR first hyperpolarizability average of H_2O_2 using LPNO-CCSD/aug-cc-pVDZ as a function of T_2 ratio

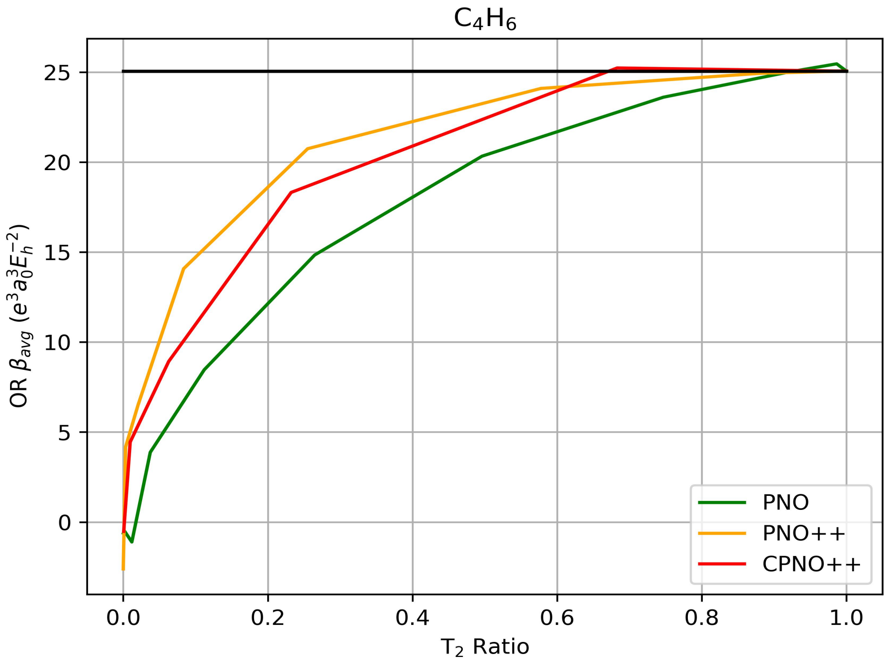

Figure S13: OR first hyperpolarizability average of C_4H_6 using LPNO-CCSD/aug-cc-pVDZ as a function of T_2 ratio

The relationship between the T_2 ratio and cutoffs along with the data for generating the figures can be found here: https://doi.org/10.5281/zenodo.14014236

References

(S1) Johnson, R. D. `http://cccbdb.nist.gov/`, NIST Computational Chemistry Comparison and Benchmark Database, NIST Standard Reference Database Number 101 Release 22, May 2022.
